# Assessment of Hypoxia Inducible Factor Levels in Cancer Cell Lines upon Hypoxic Induction Using a Novel Reporter Construct

**DOI:** 10.1371/journal.pone.0027460

**Published:** 2011-11-23

**Authors:** Wenyu Zhou, Timothy L. Dosey, Travis Biechele, Randall T. Moon, Marshall S. Horwitz, Hannele Ruohola-Baker

**Affiliations:** 1 Department of Biology, University of Washington, Seattle, Washington, United States of America; 2 Department of Biochemistry, University of Washington, Seattle, Washington, United States of America; 3 Department of Pharmacology, University of Washington, Seattle, Washington, United States of America; 4 Department of Pathology, University of Washington, Seattle, Washington, United States of America; 5 Institute for Stem Cell and Regenerative Medicine, University of Washington, Seattle, Washington, United States of America; Northwestern University, United States of America

## Abstract

Hypoxia Inducible Factor (HIF) signaling pathway is important for tumor cells with limited oxygen supplies, as it is shown to be involved in the process of proliferation and angiogenesis. Given its pivotal role in cancer biology, robust assays for tracking changes in HIF expression are necessary for understanding its regulation in cancer as well as developing therapies that target HIF signaling. Here we report a novel HIF reporter construct containing tandem repeats of minimum HIF binding sites upstream of eYFP coding sequence. We show that the reporter construct has an excellent signal to background ratio and the reporter activity is HIF dependent and directly correlates with HIF protein levels. By utilizing this new construct, we assayed HIF activity levels in different cancer cell lines cultured in various degrees of hypoxia. This analysis reveals a surprising cancer cell line specific variation of HIF activity in the same level of hypoxia. We further show that in two cervical cancer cell lines, ME180 and HeLa, the different HIF activity levels observed correlate with the levels of hsp90, a cofactor that protects HIF against VHL-independent degradation. This novel HIF reporter construct serves as a tool to rapidly define HIF activity levels and therefore the therapeutic capacity of potential HIF repressors in individual cancers.

## Introduction

Hypoxia is well known to fundamentally regulate many aspects of cell biology. Most of the effects of hypoxia involve the hypoxia inducible factor (HIF), a highly conserved and crucial oxygen regulated heterodimeric transcription factor composed of an alpha (α) and a beta (β) subunit. Both of these subunits belong to the PER-ARNT-SIM (PAS) group in basic-helix-loop-helix (bHLH) family of transcription factors [1]. Two genes encoding mammalian HIFα subunits (HIF1α, HIF2α) are well studied: HIF1α is ubiquitously expressed whereas HIF2α exhibit more restricted tissue distribution [2]. In normoxia, HIFα undergoes prolyl hydroxylation and binds to an ubiquitin E3-ligase, the von Hippel-Lindau (VHL) protein, which leads to polyubiquitination and rapid proteosomal degradation of HIFα [3], [4]. Under hypoxia, HIFα hydroxylation is inhibited, resulting in accumulation of HIFα and formation of HIFα-HIFβ heterodimers. The heterodimers further complex with the coactivator p300, and bind to the promoters of HIF target genes to induce gene expression [5]. In addition to hypoxia, many other pathways can affect HIF stabilization [6], [7]. Cofactors, such as PACF P300/CBP associated factor [8] and hsp90 [9], [10], also help facilitate HIF stabilization and enhance HIF activities. Hsp90 has shown to protect HIFα against VHL-independent degradation that can occur in hypoxia [11].

The well studied HIF target genes include those that are involved in oxygen delivery and cell proliferation, such as the vascular endothelial growth factor (*VEGF*) [12], [13] and *p21* [14]. In addition, HIF facilitates adaptation to oxygen deprivation by regulating genes involved in glucose uptake and metabolism, such as carbonic anhydrase (*CA9*) [15], which maintains cellular pH_i_ homeostasis under hypoxia. It was recently reported that HIF and its target gene, *Oct4*, are responsible for hypoxia induced cancer stem cell phenotype that is thought to drive the progression and aggressiveness in certain tumors [16]. Given its pivotal role in angiogenesis and tumor progression, HIF is a therapeutically attractive target and blocking HIF, especially when combined with conventional therapies, has shown beneficial effects [17], [18].

To examine HIFs' temporal and spatial expression in tissues, several direct and indirect reporter systems are developed in order to track HIF protein expression *in vivo*. Either full length HIF cDNA, or a fragment under oxygen-dependent regulation has been linked to fluorescent protein [19], or firefly luciferase [20] for constructing HIF-fusion proteins. Alternatively, since HIF fusion protein studies do not reveal whether HIF complex is transcriptionally active, promoter based reporters have also been developed. Typically, 5–8 repeats of the hypoxia response elements (HREs) (5′-GCCCTACGTGCTGTCTCACACAGC-3′) from the 3′ enhancer region of human Epo gene, or the HRE from VEGF (5′-CACAGTGCATACGTGGGCTCCAACAGGTCCTCT-3′) are linked in tandem with a minimal promoter to drive the expression of a downstream reporter gene [21], [22]. It is worth noting that in these constructs, HRE contains not only the HIF-1α or HIF2α consensus binding sites (5′-CGTG-3′ and 5′-TRCGTG-3′, respectively), but also *Epo* or *VEGF* promoter specific sequences. Recently *Oct4* has shown to be induced by HIF under hypoxia [16], however, *Oct4* promoter only contains three repeats of CGTG, the actual HIF binding site but not the HRE sequences observed either in *Epo* or *VEGF* promoter.

In order to maximize the specificity and sensitivity of the reporter construct, a strategy of using the most primitive transcription factor binding site in tandem in a reporter has been successfully utilized previously in the case of Wnt-pathway analysis [23], [24]. In the present study, we utilized a similar strategy to build up a promoter based reporter, only incorporating the minimal HIF1α and HIF2α binding sites together (CGTGTACGTG) in tandem in the promoter. We show that this new HIF reporter with HIF binding repeats (HBR) has a good signal to background ratio and signal dynamics in deoxygenation and reoxygenation. We also demonstrate that the signal is HIF dependent as revealed by HIF RNAi studies, and it correlates with the cellular HIF protein level in different cell lines. By utilizing our new construct, we show that HIF activity levels vary significantly in different cancer cell lines cultured in the same degree of hypoxia. We further reveal that in two cervical cancer cell lines, the differences in HIF activity levels correlate with the level of the HIF cofactor, Hsp90, which protects HIF against VHL-independent degradation.

## Results

**Figure 1:**
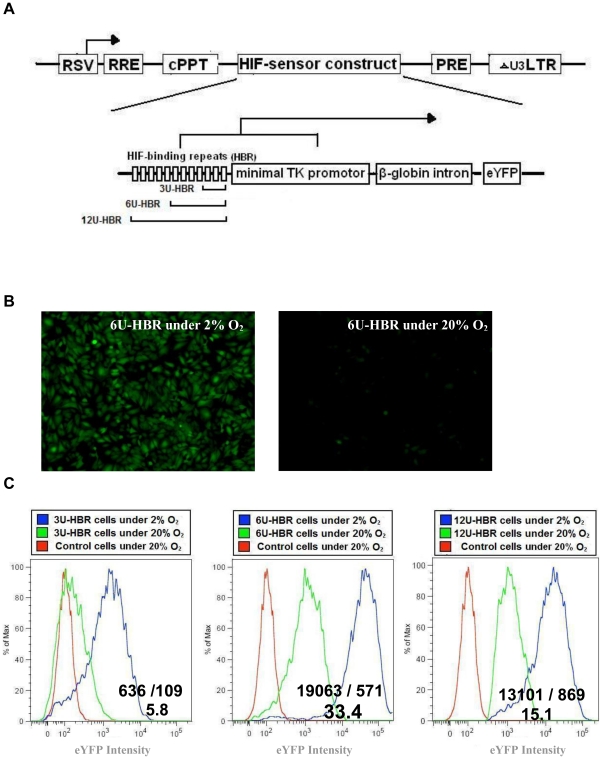
Schematic representation of HIF-HBR reporter construct. 3, 6 or 12 tandem units of HIF binding repeats (3U, 6U or 12U-HBR) and a TK minimal promoter together regulate the eYFP expression. b–c) Optimization of the number of HIF binding repeats. b) 6U-HBR HeLa cells turn eYFP on in 2% but not in 20% oxygen. Equal amount of cells (5×10^4^) were plated in both culture dishes and microscopy and FACS analysis were performed after 2 days; c) the signal-to-background ratio is represented by FACS analysis. HeLa cells were transduced with HIF-HBR reporter lentiviral particles for 24 hrs and then grown under normoxia (20% O2) or hypoxia (2% O2) for 48 hours before FACS analysis. Ratios are of geometric means of eYFP intensity in 2% to 20% oxygen.

We constructed a lentiviral plasmid, in which the expression of enhanced yellow florescent protein (eYFP) was under the regulation of twelve tandem repeats of minimal HIF-binding sites (CGTGTACGTG), followed by a minimal human thymidine kinase (TK) promoter (12U-HBR). Also, a 770 bps of β-globin intron sequences was incorporated between the TK promoter and eYFP for better transcription of eYFP (Figure 1a, Figure S1). To assay the construct's function *in vitro*, we transduced the virus into HeLa cells and cultured them in either normoxic (20% O_2_) or hypoxic (2% O_2_) condition. After 24 hours, we observed that cells under hypoxia uniformly expressed eYFP (∼70% of eYFP positive), while those under normoxia only gained fluorescence with greatly reduced intensity (∼6% of eYFP positive, Figure 1b and Figure S2).

To further optimize the signal to background ratio, we reduced the number of HIF-binding sites, thereby creating HIF-reporters with 3 tandem binding sites (3U-HBR) or with 6 sites (6U-HBR) (Figure 1a). When analyzing in HeLa cells, we observed that 6U-HBR construct achieved a better signal-to-background ratio than 3U-HBR and 12U-HBR constructs (33.4, 5.8 and 15.1, respectively. Figure 1c). To compare the specificity and sensitivity to other hypoxia reporters, we specifically obtained one of the widely used HIF reporter 5HRE_hCMV_Luc [25]. After transient transfected 5HRE_hCMV_luc reporter in HeLa cells, we observed 50–100 fold greater luciferase activity under 2% O_2_ as compared to cells growing in 20% O_2_, which was consistent with the original finding. In comparison, our HBR_eYFP reporter shows about 30–40 fold increase in 2% O_2_, an increase that falls in a similar scale as seen with 5HRE_hCMV_luc reporter. With the similarity in signal intensity, however, the two HIF reporter constructs are not directly comparable, given that they have distinct backbone sequences, different minimal promoters and reporter genes, all of which could render them distinct behavior and dynamics under hypoxia. In this study, 6U-HBR was used for further optimization and characterization.

**Figure 2:**
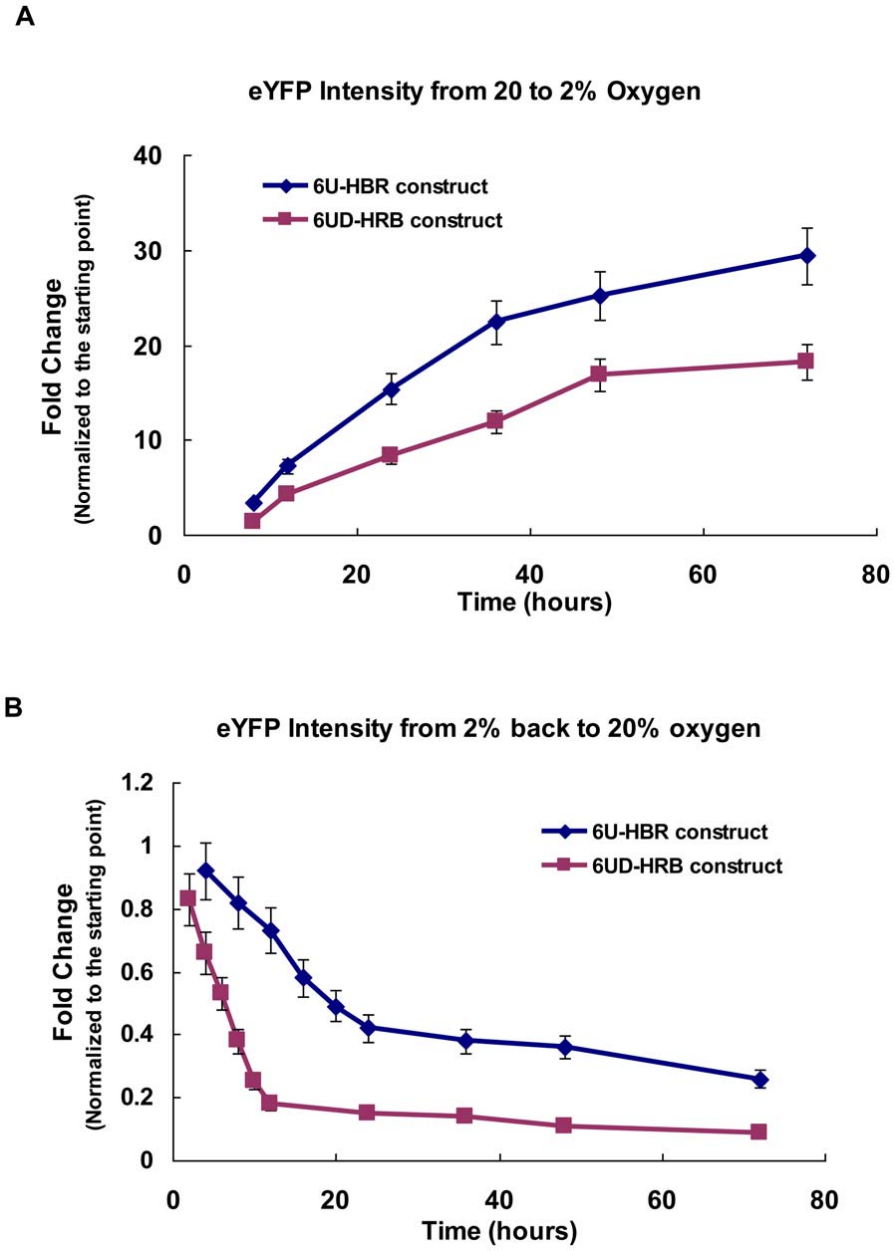
Dynamics of 6U-HBR and 6UD-HBR HeLa cell lines. 5×10^4^ cells were plated onto 35 mm plates and then cultured under either normoxia (20% O_2_) or hypoxia (2% O_2_). At different time points, cells were harvested and fixed for FACS analysis. Mean and error bars are from three biological repeats.

We next characterized the dynamics of the construct in the process of deoxygenation and reoxygenation. When transferred from normoxia to hypoxia (2%), 6U-HBR-HeLa cells had rapid response (Figure 2a); however, when transferred from hypoxia back to normoxia, the fluorescent intensity decreased slowly (72 hours to reach the basal level; Figure 2b). For a construct with better ability to timely reflect the turnover of HIF, we modified eYFP and generated a destabilized version by attaching mouse ornithine decarboxylase 422–461 domain, a region responsible for rapid degradation of the protein [26], to the C-terminus of eYFP, therefore creating the construct of 6U-destabilized-HBR (6UD-HBR). We observed that 6UD-HBR had a reduced fluorescence but similar dynamics as seen with 6U-HBR (Figure 2a). When transferred back to normoxia, the fluorescent signal of 6UD-HBR cells showed dramatic reduction reaching the lowest level in 10 hours. Based on these observations, 6U-HBR is more sensitive in detecting changes in the process of deoxygenation while 6UD-HBR better reflects changes in the process of reoxygenation.

**Figure 3:**
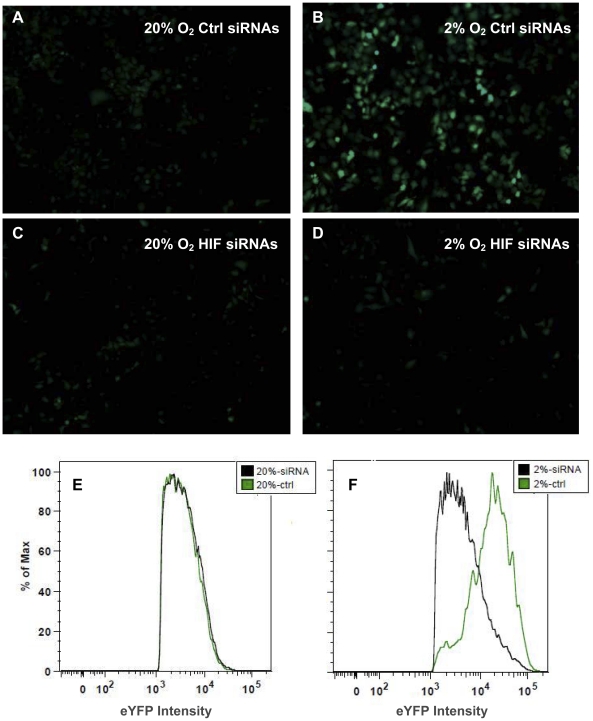
6U-HBR construct is HIF-dependent. 6U-HBR HeLa cells transfected with siRNAs against three HIFs (HIF1α, HIF1β and HIF2α) are eYFP negative under hypoxia, demonstrated by both microscopy (upper panel a–d) and FACS results (lower panel e and f). Equal amount of cells (5×10^4^) were plated in culture dishes and microscopy and FACS analysis were performed after 2 days after siRNA transfection.

**Figure 4:**
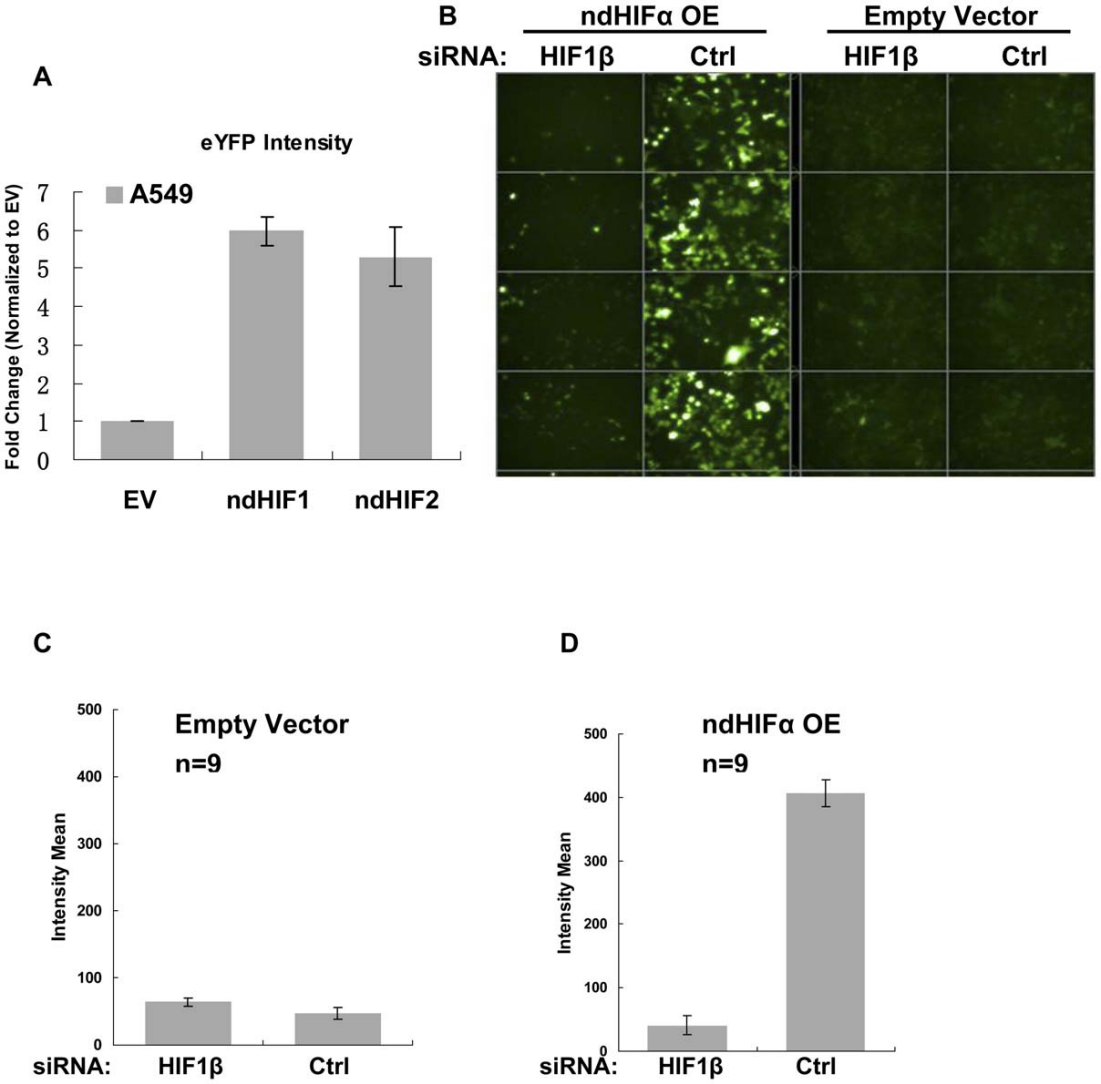
Sensitivity of 6U-HBR construct. a) 6U-HBR construct in A549 is activated either with ndHIF1α or ndHIF2α, but not with empty vector construct. b–d) HIF1β siRNA alone largely reduces fluorescent signals induced by ndHIF1α/ndHF2α over-expression (ndHIFα OE) in HeLa cells, indicating that the eYFP signals are reversible by manipulating levels of HIF factors. Both microscopy images (b) and fluorescent quantification (c and d) are shown.

To confirm HIF-dependency of the reporter, we performed RNAi experiments targeting HIFs under hypoxia. When knocking down three HIFs (HIF1α, HIF1β and HIF2α), 6U-HBR-HeLa cells under hypoxia had greatly reduced fluorescence, close to the level seen under normoxia (Figure 3a–f, approximately equal numbers of cells observed in a–d). Furthermore, we showed that over-expression of non-degradable HIF1α or HIF2α alone was sufficient to activate the expression of the construct (Figure 4a and 4b). To test whether the signal induced by HIFα over-expression (OE) was directly caused by the canonical activity of HIFα, we introduced RNAi against the essential cofactor of HIFα, HIF1β, in HIFα OE cells. We showed that when transfected with HIF1β RNAi, the eYFP intensity induced by HIFα OE was greatly reduced (Figure 4b–d). It is worth noting that in the RNAi experiments performed in a high-throughput platform, cells showed equivalent intensity across wells in the same treatment group (Figure 4b–d). These experiments demonstrate the applicability and sensitivity of the 6U-HBR construct in a high-throughput platform. The sensitivity of the construct to both HIF1α and HIF2α was further confirmed by the results when knocking down individual HIFα or HIFs with various combinations in HeLa cells (Figure S3). Altogether, we show that the reporter is directly sensing HIF signaling in the cells.

**Figure 5:**
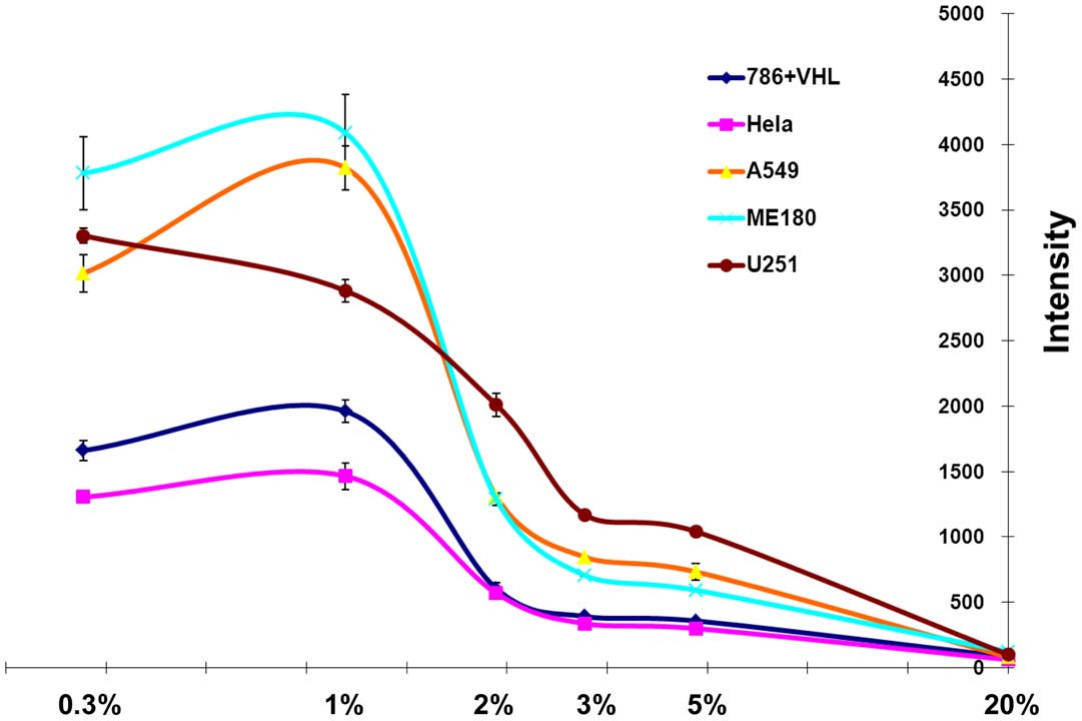
Cancer cells with 6U-HBR show different levels of HIF activity under hypoxia. Five cancer cell lines infected with 6U-HBR lentivirus were cultured under various degree of hypoxia, including 20%, 5%, 3%, 2%, 1% and 0.3% of O_2_. Their eYFP intensities were determined after 4 days in culture by FACS analysis. The degrees of hypoxia are presented as log of relative O_2_ level to normoxia (20% O_2_).

In mammalian embryogenesis and development, tissues develop in a microenviroment with various levels of oxygen. It has been shown that hypoxia, on one hand, is essential for heart formation [27], [28], [29] and endochondrial bone formation [30], [31], [32], [33]; however, on the other hand, it impairs adipose tissue development [34], [35] and skin myofibroblast differentiation [36]. Given the critical role of HIF signaling in hypoxia, one fundamental question is whether cells gain tissue-specific responses to their low oxygen environment by differentially regulating HIF pathway. To determine how cells from different tissue origins respond to hypoxia in terms of HIF levels and activities, we examined 5 6U-HBR carcinomas cell lines, namely, 786+VHL from renal cell adenocarcinoma, A549 from lung carcinoma, ME180 from epidermoid carcinoma in cervix, U251 from glioma, and HeLa from cervical adenocarcinoma. Cells were incubated in different oxygen levels (20%, 5%, 3%, 2%, 1% or 0.3%) for 4 days, and the average 6U-HBR fluorescence was determined by FACS analysis. These cells might have been expected to behave similarly under hypoxic conditions due to the fact that they have adapted to culture conditions; however, we observed that they responded distinctly, under the same degree of oxygen. For instance, ME180 and HeLa both had little response at 3% of oxygen, however, when oxygen level was below 3%, ME180 exhibited dramatic increase of the signal, while HeLa had relatively low increase (Figure 5). On the other hand, the signal of U251 increased slowly at 5% and 3% of oxygen, more dramatically in 2% and 1% of oxygen, and distinctly continued to increase even in 0.3% of oxygen (Figure 5).

**Figure 6:**
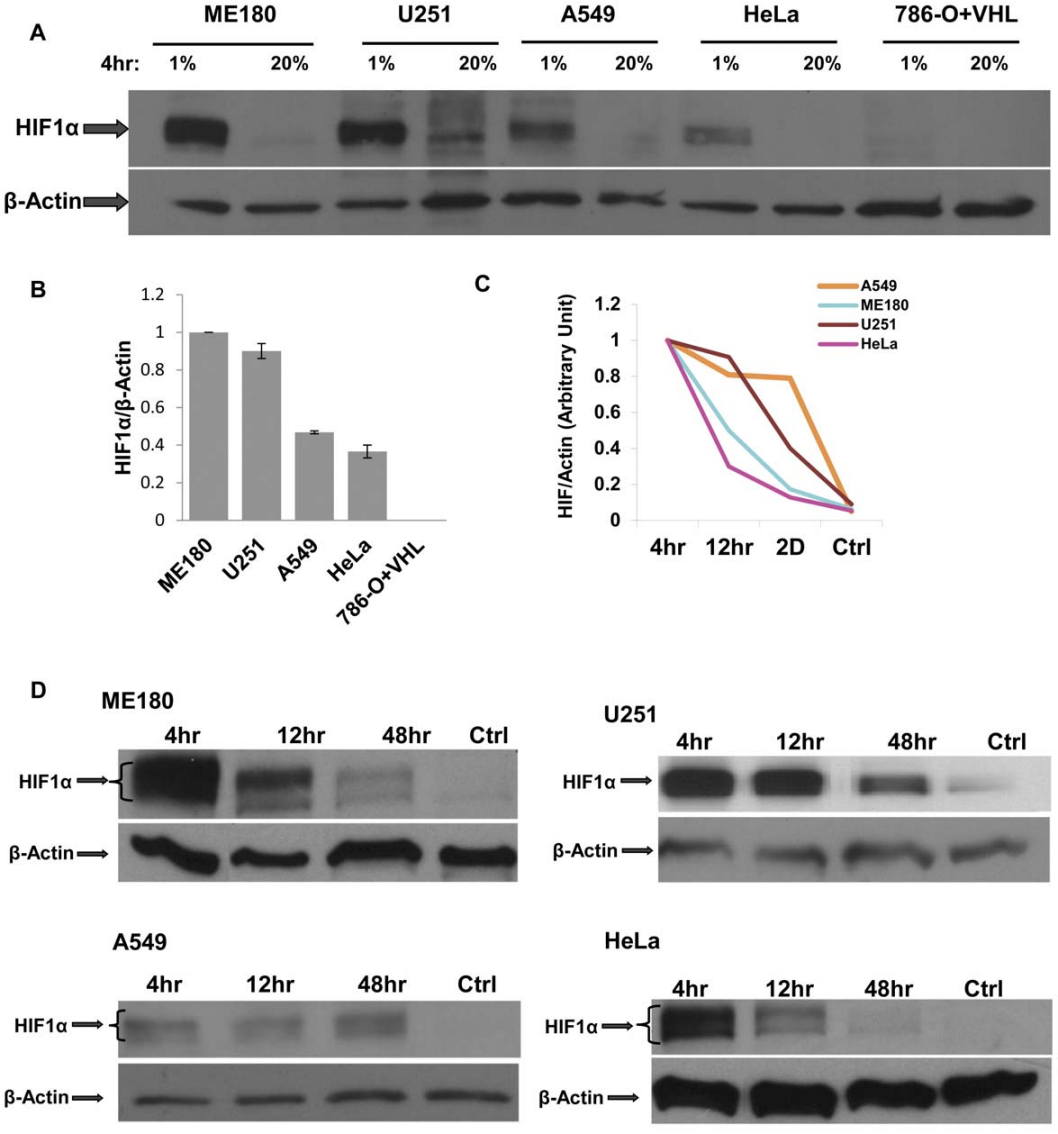
HIF protein levels vary in different cancer cells under 1% O_2_. a) HIF1α in ME180, U251, A549, HeLa and 786-O+VHL cells under 1% O_2_ for 4 hours. b) Quantification for HIF1α protein shown in a). Quantification was performed on scanned film images obtained from separate western blots. c) HIF1α levels and d) quantification in ME180, U251, A549, HeLa and 786-O+VHL cells under 1% O_2_ at three time points: 4 hours, 12 hours and 48 hours, compared to the levels under 20% O_2_ as controls.

We further verified that 6U-HBR signals in these cell lines correlated with intracellular HIF protein levels. Since the 1% oxygen environment exhibited the greatest difference in 6U-HBR signals among ME180, U251 and HeLa cells, we used this level of oxygen to assay HIF mRNA and protein. While HIF1α mRNA levels were similar among these cell lines, HIF2α mRNA levels varied (Figure S4), which was in accordance with a previous study [2]. For protein level, we first showed that four hours in hypoxia induced higher HIF1α protein levels in ME180, U251 and A549 than in HeLa and 786-O+VHL (Figure 6a–b). Since it has been previously reported that in prolonged hypoxia, HIF1α protein degrades while HIF2α exhibits minimal change [37], we hypothesized that not only the total HIF protein levels but also the stabilization dynamics of the proteins are responsible for different 6U-HBR reporter activities in these cell lines. We therefore analyzed the differences in degradation kinetics of HIFα proteins in all the five cancer cell lines in 1% of oxygen. This analysis revealed that HIF1α in HeLa cells is less stable during prolonged hypoxia than in any other cell types analyzed (Figure 6c and d). When comparing HIF2α levels in 1% hypoxia versus normaxia, the difference among these five cell lines is diminished in terms of total protein levels and degradation patterns (Figure S5). However, while no HIF1α protein was observed in 786-O+VHL cells, HIF2α protein was induced in hypoxia in this cell lines, explaning the responsiveness of this cell line to the 6U-HBR reporter (Figure S5; Figure 5). Altogether, the data show that higher levels of more stable HIFα proteins are observed in ME180, A549 and U251 compared to HeLa and 786-O+VHL cells, supporting the findings revealed by 6U-HBR reporter that these five cell lines display distinct HIF activities in the same degree of hypoxia.

**Figure 7:**
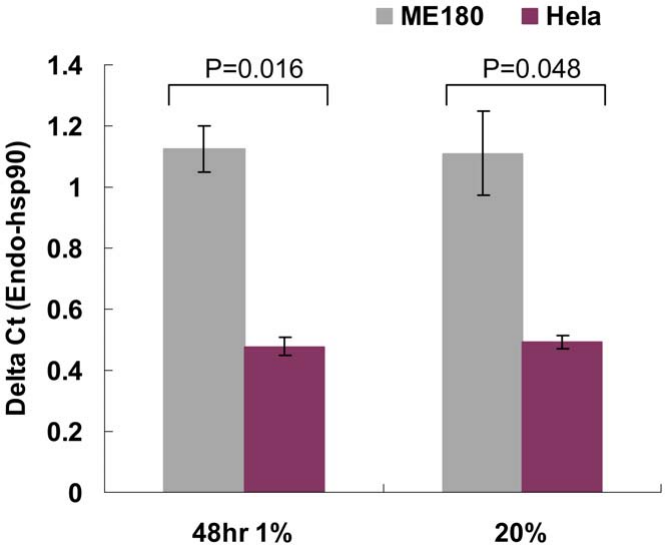
Hsp90 mRNA levels are different in ME180 and HeLa both under hypoxia and normoxia. Cells were either incubated under 1% hypoxia or 20% normoxia for 48 hours before quantification the hsp90 mRNA level by real-time PCR. The error bars are presented as sample error of the mean (SEM) from three independent biological experiments.

**Figure 8:**
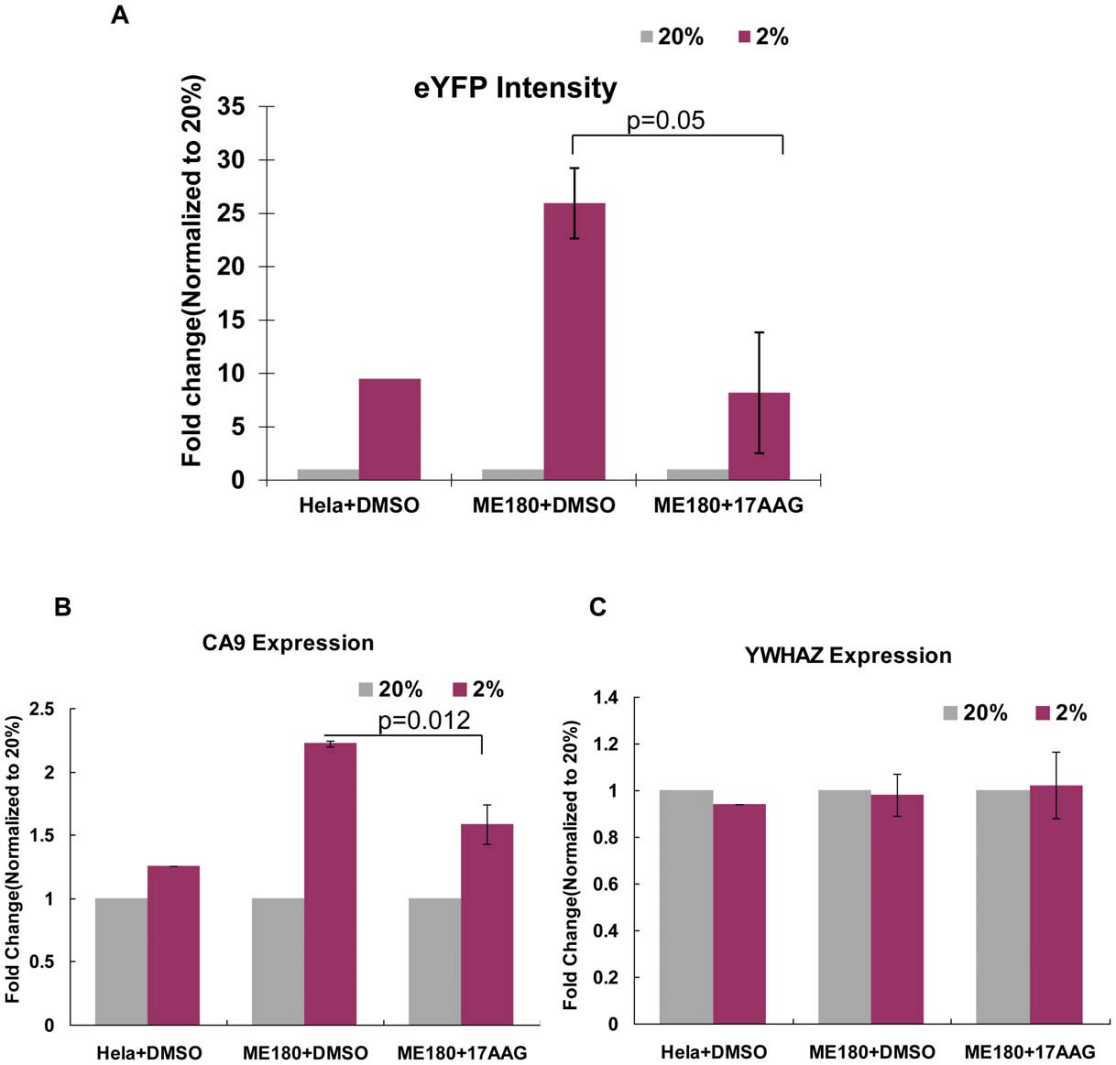
17-AAG reduces the difference in HIF levels and its transcriptional activity between ME180 and HeLa. Results of three independent experiments are summarized and the error bars are presented as sample error of the mean (SEM). a) Fluorescence intensities of HeLa in DMSO, ME180 in DMSO and ME180 in 100 nM 17-AAG in 2% hypoxia are normalized to that in 20% normoxia. b) The fold changes of CA9 mRNA levels normalized to 20% normoxia in those cells suggest that the difference, in terms of HIF activity, is reduced in the presence of 17-AAG. c) The stable expression of another housekeeping gene, tyrosine 3-monooxygenase/tryptophan 5-monooxygenase activation protein, zeta polypeptide (YWHAZ), demonstrates the generally normal transcriptional activities in those cells treated either with DMSO or 17-AAG.

We chose HeLa and ME180 for further study since they are both cervical cancer cell lines that exhibit very distinct responses to hypoxia. In order to understand the regulation of HIFα in these two cervical cancer cell lines, we analyzed mRNA microarray data previously performed in these cell lines in 2% hypoxia [38]. Interestingly, we identified hsp90 mRNA differentially expressed between HeLa and ME180. We first validated this finding by real-time PCR analysis and showed that ME180 has higher levels of hsp90 both in normoxia and hypoxia (Figure 7). To determine whether higher levels of hsp90 in ME180 could be causal for higher HIF activity, we reduced hsp90 binding to HIF1α in ME180 by incubating cells with 17-allylamino-demethoxygeldamycin (17-AAG) in 2% hypoxic conditions. 17-AAG inhibits ATP binding to hsp90, thereby preventing the interaction of hsp90 and its target protein [39]. Since hsp90 interaction with HIF1α is shown to increase HIF1 stability in hypoxia [11], 17-AAG should reduce hsp90 dependent HIF1 activity. Accordingly, 6U-HBR ME180 incubated with 17-AAG showed lower levels of 6U-HBR reporter activity than observed in the controls (Figure 8a). Furthermore, the mRNA expression of HIF1α target gene CA9 was significantly reduced (Figure 8b), suggesting that HIF activity was reduced in ME180 when hsp90 was rendered inactive. These data support the hypothesis that the difference in hsp90 levels is causal for the difference in HIF activity observed in the two cervical cancer cell lines. We excluded the possibility of reduction of HIF activities caused by general transcriptional regulation in the cells by showing stable expression of another endogenous housekeeping gene in both cell types (WYHAZ, Figure 8c).

## Discussion

Here we create and characterize a novel HIF reporter construct, in which eYFP expression is regulated by tandem repeats of minimal HIF1α and HIF2α binding sites. By employing cells that are infected with lentivirus of the HIF-HBR construct, we show that cells with 6 tandem repeats as the promoter in the construct (6U-HBR) give better signal-to-background ratio than those with 3 or 12 repeats. Also, in terms of reporter kinetics, the 6U-HBR construct achieves a stronger signal in the process of deoxygenation, while cells with 6U-destabilized-HBR construct more accurately reflect the reduction of HIF levels in the process of reoxygenation. Moreover, through siRNA studies against HIF1α, HIF2α and HIF1β as well as over-expression studies of ndHIF1α and ndHIF2α, we demonstrate that the construct is HIF specific and is able to respond to both HIF1α and HIF2α. Utilizing 5 different cancer cell lines infected with 6U-HBR construct, we observe that different cancer cell lines have distinct eYFP signals under the same oxygen levels, which correlate with the total amount of HIF1 α and HIF2 α proteins in these cell lines.

We utilized the reporter to explore difference in the hypoxic response of HeLa and ME180 cervical cancer cell lines. We observed that these two cancer cell lines have distinct 6U-HBR signals which correlate with the amount of HIFα proteins in response to the same level of hypoxia. Similarly, the different behaviors between HeLa and ME180 under hypoxia are also observed in angiogenic growth factor expression [40], [41] as well as in proteasome, histone deacetylase [42] and Hsp90 action [43]. Among them, Hsp90 is of particular interest. In accordance to previous analysis [43], we show that ME180 has higher levels of Hsp90 mRNA than HeLa both under normoxia and hypoxia. Higher basal level of HIF cofactor Hsp90 in ME180 could contribute to the observed higher HIF activity, since hsp90 binding to HIF has been reported to protect HIFα against VHL-independent degradation [9], [11]. Low oxygen levels inactivate PHD/VHL dependent degradation of HIF, resulting in stabilized and active HIF transcription factor. However, HIFα is eventually degraded in hypoxic conditions. This VHL-independent degradation of HIF1α has shown to be RACK1 dependent [11]. Hsp90 competes with RACK1 binding to HIF1α, thereby protecting HIF1α against hypoxic degradation. In present study, we show that ME180 cells have higher levels of hsp90 and more stable HIFα proteins compared to HeLa (Figure 6). We further show that reduction of hsp90 in ME180 diminishes the difference of HIF level and activity as compared to HeLa. These data suggest that different Hsp90 levels could be causal for the different HIF activity levels observed in the two cervical cancer cell lines under the same level of hypoxia. It will be interesting to test in the future whether oxygen/PDH/VHL-independent HIFα degradation is in general the key regulator of the HIF activity differences observed in cancer cell lines under the same level of hypoxia.

It is known that cervical cancer cells are often infected with an oncogenic-type human papillomavirus (HPV), and HeLa is transformed by HPV18 while ME180 by HPV68. HPV of different types vary in the expression and regulation of the two primary HPV oncogenes, E6 and E7 [44], that are sufficient to alter HIF-1α level. HPV11 E6 and HPV31 E6 can stimulate HIF-1α expression as a consequence of ubiquitin-dependent degradation of p53 [45] while HPV16 E7 can interact with a cullin-2 ubiquitin ligase complex that mediates VHL-dependent HIFα degradation [46]. It will be interesting to reveal in the future whether different responses to hypoxia observed in HeLa and ME180 are associated with the type of oncogenic HPV they are infected with.

In this study we report a novel HIF reporter construct containing tandem repeats of minimum HIF binding sites upstream of eYFP coding sequence. We show that the signal of the reporter is HIF dependent and correlates with the cellular HIF protein levels in different cell lines. By utilizing this new construct, we find a surprising variation of HIF activity in different cancer cell lines under the same level of hypoxia. This novel HIF reporter construct may serve as a tool to rapidly define HIF activity levels and therefore therapeutic capacity of potential HIF repressors in individual cancers.

## Materials and Methods

### Cells, tissue culture and hypoxia induction

HeLa and ME180 (cervical carcinoma), A549 (lung carcinoma), U251 (glioma) cells were from the American Type Culture Collection (Rockville, MD). 786-O cells transfected with a wild-type VHL were obtained from Dr. William G. Kaelin Jr. (Dana-Farber Institute, Boston, MA). Cancer cells were grown in Dulbecco's modified Eagle's medium (DMEM) supplemented with penicillin, streptomycin and 10% fetal bovine serum (FBS, Invitrogen, Carlsbad, CA). Cells were passed with Trypsin/EDTA (Invitrogen, Carlsbad, CA) when they reached 80% confluence.

### Hypoxia induction

Cells were cultured in multi-gas incubators (Sanyo, San Diego, CA). Nitrogen gas was supplied to the chambers in order to induce a controlled reduced percentage of oxygen. For normoxia, cells were cultured in incubators containing 5% CO_2_ and atmospheric concentration of O_2_, approximately 20% to 21% O_2_. Throughout this paper “normoxia” was referred as 20% O2.

### Lentiviral plasmid construction

We generated HIF reporter construct with HIF1α consensus binding site CGTG followed HIF2α site TACGTG together as one unit of binding site for HIFα factors and repeated them in tandem 12 times (12U-HBR), with 5 base pairs of nucleotides of various combinations spacing between. Induction element CACAG, a DNA element necessary for hypoxic induction, was evenly inserted into the whole sequences three times to assist in the induction process. The final HIF-binding sequences were directly synthesized by Integrated DNA Technologies, Inc, Coralville, Iowa (Figure S1). TK minimal promoter followed by 770 bps β-globin intron sequences and eYFP was amplified from pBARL plasmid (courtesy of Dr. Randall Moon laboratory, University of Washington, Seattle, WA) and linked to HIF-binding sequence by fusion PCR. The HIF-TK-eYFP sequences were further ligated into a lentiviral plasmid pRRL-cPPT-X-PRE-SIN [47] (courtesy of Dr. William Osborne laboratory, University of Washington, Seattle, WA) between ClaI and XhoI sites. 6U-HBR and 3U-HBR were constructed in the same way, except the HIF-binding sequences only contain six binding repeats (6U-HBR) or three repeats (3U-HBR). To build up 6U-HBR-destabilized version, the mouse ornithine decarboxylase 422–461 domain sequences were first amplified from Plasmid M38 TOP-dGFP (courtesy of Dr. Randall Moon laboratory) and then inserted into 6U-HBR construct through MluI site following the 3′ of eYFP sequence.

### Lentivirus production and stable transduction of cell lines

Lentiviral plasmids were transfected into FT293 cell lines (Invitrogen, Inc, Carlsbad, CA) by calcium phosphate transfection to produce lentiviral particles. Specifically, transferring plasmid (12U-HBR, 6U-HBR, 3U-HBR, or 6U-HBR-destabilized construct), packaging plasmid, VSVG plasmid and REV plasmid were transfected together at 23∶15∶8∶11.5 ratio, and 25 µg of mixed plasmids were transfected per 150 mm plate with 1 mL of 2X HBS and 0.1 mL 2.5 M CaCl_2_. After transfection, media were changed after 24 hours and viral supernatants were harvested and filtered after 72 hours. To obtain concentrated virus, the viral supernatants were further centrifuged at 6100 rpm for 17 hours at 4°C. Precipitate was resuspended in 1X TBS (50 mM Tris.HCl, pH 7.4 and 150 mM NaCl) and then stored in −80°C before use. To transduce cell lines with virus, appropriate amount of virus were directly added into the media with the presence of hexadimethrine bromide at 4 ng/ml (Polybrene, Invitrogen Inc, Carlsbad, CA) and media was changed after 24 hours.

### Fluorescence-activated cell sorting (FACS) analysis

Cells to be analyzed were trypsinized from plates, centrifuged down and then fixed in 4% of paraformaldehyde for at least 30 minutes before the analysis. Standard settings were applied in cell sorting with appropriate channel voltages and at least 30,000 cells were analyzed for each experiment. FITC channel was used to detect eYFP fluorescence. FlowJo (Tree Star, Inc. Ashland, OR) was employed to visualize data and FITC values were defined as the geometric mean of florescence intensity of eYFP positive population for hypoxic samples. For samples incubated in 20% O_2_, the intensities were defined as the geometric mean of florescence intensity of eYFP of the total population (eYFP negative).

### Non-degradable HIF (ndHIF) over expression

To obtain constitutively stable expression HIFα protein, ndHIF over-expressing plasmids (Addgene plasmid 19005 and 19006) were used, in which two the Proline sites of HIF cDNA were changed to Alanine as described previously [48]. Retrovirus made from those plasmids were infected into HeLa and A549 cell lines in the presence of hexadimethrine bromide at 4 ng/ml (Polybrene, Invitrogen Inc. Carlsbad, CA) and media was changed after 24 hours. Over-expression of HIF1α and HIF2α was confirmed by western blots as shown in Figure S6.

### siRNA against HIF assay

siRNAs against HIF factor were gift of Dr. Zhan Zhang [38]. siRNAs were transient transfected into cells on 6-well plate with Lipofectamine 2000 (Invitrogen Inc. Carlsbad, CA) following the Lipofectamine 2000 instruction. Normally, ∼60% reduction of gene expression is obtained after two days (Figure S3b). Specifically, siRNAs and Lipofectamine were transfected at 100 pmol: 2.5 µl per well. Media was changed after 24 hours and cells were cultured under hypoxia thereafter. Cells were fixed for FACS after 24 hours in hypoxia. In the high through-put experiment, 6U-HBR HeLa cells stably expressing ndHIF1α/ndHIF2α (ndHIFα OE) or empty vector (EV) were plated in 384-well plate. HIF1β siRNA or scramble siRNA was transfected into ndHIFα OE cells and EV cells in 384-well plate using RNAiMAX reagent (Invitrogen Inc, Carlsbad, CA). Operetta high content imaging system (PerkinElmer, San Jose, CA) was used for microscopy images and fluorescence quantification.

### HIF protein western blots

For Western blotting analysis, cells were washed with DPBS and directly lysed on culture dish using homogenizing buffer consisting of 20 mM Tris-HCl (pH 7.5), 150 mM NaCl, 15% Glycerol, 1% Triton, 3% SDS, 25 mM β-glycerolphosphate, 50 mM NaF, 10 mM NaPyrophosphate, 0.5% Orthovanadate, 1% PMSF (all chemicals are from Sigma-Aldrich, St. Louis, MO), 25 U Benzonase® Nuclease (EMD Chemicals, Gibbstown, NJ) and protease inhibitor cocktail (Complete Mini, Roche Applied Science, Germany). The protein concentration of each sample was determined by BCA protein assay system (Thermo Scientific, Rockford, IL). 20 µg of protein extracts were loaded, separated by 7.5% SDS-PAGE, and transferred to polyvinylidene difluoride membranes (Hybond-N+, Amersham Pharmacia Biotech, Buckinghamshire, England). Membranes were blocked with 5% nonfat dry milk for at least 60 minutes at room temperature, and incubated overnight at 4°C with HIF1α antibody (BD biosciences, San Jose, CA) diluted at 1∶2000, or HIF2α antibody (Abcam, Cambridge, MA) diluted at 1∶1000 (or HIF2α antibody from Novus Biologicals diluted at 1∶5000). Finally, after blots had been incubated for 1 hour with horseradish peroxidase-conjugated secondary antibodies, they were visualized by enhanced chemiluminescence (Millipore Corp, Billerica, MA). Protein expression levels in the gel were quantified by densitometry implemented in Image-J (National Institutes of Health, Bethesda http://rsb.info.nih.gov/ij/, 1997–2009).

### mRNA quantification among different cell lines

RNA was extracted from cells adherent on plates using Trizol reagent (Invitrogen Inc. Carlsbad, CA) and cDNA was synthesized with Omniscript RT kit (Qiagen Inc. Valencia, CA). To determine the normalization factor, geNorm algorithm was used to seek appropriate endogenous housekeeping genes for comparison among different cell lines [49]. HPRT1 and YWHAZ were chose as the endogenous control genes for normalization. mRNA level of HIF1α, HIF2α, Hsp90 and endogenous control genes were quantified in triplicates of 20 µl of PCR reactions using SyberGreen system (Applied Biosystems, Foster City, CA), each with 25 ng cDNA. All of the reactions were performed in 7300 real time PCR system (Applied Biosystems, Foster City, CA) using default settings. The following primers were used in the quantification: *HIF1α_*F: 5′-TCCATGTGACCATGAGGAAA-3′ and *HIF1α_*R: 5′-CCAAGCAGGTCATAGGTGGT -3′; *HIF2α_*F: 5′-CCACCAGCTTCACTCTCTCC-3′ and *HIF2α_*R: 5′-TCAGAAAAGGCCACTGCTT-3′; *hsp90*_F: 5′-TCTGGAAGATCCCCAGACAC-3′, *hsp90*_R: 5′- AGTCATCCCTCAGCCAGAGA-3′.

### Knockdown of hsp90 through 17-AAG

Cells were incubated in 2% hypoxia with 100 nM 17-AAG in media for 24 hours and then replaced by fresh media. Cells were fixed for FACS or harvested for mRNA quantification after 48 hours in hypoxia.

## Supporting Information

Figure S1 **Promoter sequence of 12U-HBR construct.** The minimal TK promoter is not shown.
(TIF)

Figure S2 **6U-HBR HeLa cells response to hypoxia as reflected by the reporter construct.** a) 6U-HBR HeLa cells turn eYFP on in 2% but not in 20% oxygen. Equal amount of cells (5×10^4^) were plated in both culture dishes and FACS analysis were performed after 2 days. The percentages of eYFP positive cells under these conditions are shown. b) The patterns of 6U-HBR HeLa cells in response to different levels of hypoxia. The percentages of eYFP positive cells and the mean intensity of each sample were shown.
(TIF)

Figure S3 **6U-HBR construct is HIF-dependent.** a) siRNAs assays against various combinations of HIFs demonstrate that the construct is HIF-dependent. HeLa cells were incubated in 2% O_2_ for one day after siRNA transfection; b) Validation is shown for knockdown experiments of HIF1β expression by HIF1β siRNAs. Mean and error bars are from three biological repeats.
(TIF)

Figure S4 **HIFα mRNA levels in ME180, U251 and HeLa cancer cell lines.** Delta Ct is calculated directly from Ct values of endogenous genes (HPRT1 and YWHAZ) minus that of HIF1α or HIF2α.
(TIF)

Figure S5 **HIF2α protein levels under 1% O_2_ in five cell lines.** a) HIF2α in ME180, U251, A549, HeLa and 786-O+VHL cells under 1% O_2_ for 4 hours. b) HIF2α levels in ME180, U251, A549, HeLa and 786-O+VHL cells under 1% O_2_ at three time points: 4 hours, 12 hours and 48 hours, compared to the levels under 20% O_2_ as controls. HIF2α antibody (Novus Biologicals,CO, NB100-122) was used.
(TIF)

Figure S6 **Over-expression of HIF1α and HIF2α by retroviral infection was confirmed in western blots.** HIF2α antibody (Abcam, Cambridge, MA, ab20654) was used.
(TIF)

## References

[1] Wang GL, Jiang BH, Rue EA, Semenza GL (1995). Hypoxia-inducible factor 1 is a basic-helix-loop-helix-PAS heterodimer regulated by cellular O2 tension.. Proc Natl Acad Sci U S A.

[2] Jain S, Maltepe E, Lu MM, Simon C, Bradfield CA (1998). Expression of ARNT, ARNT2, HIF1 alpha, HIF2 alpha and Ah receptor mRNAs in the developing mouse.. Mech Dev.

[3] Maxwell PH, Wiesener MS, Chang GW, Clifford SC, Vaux EC (1999). The tumour suppressor protein VHL targets hypoxia-inducible factors for oxygen-dependent proteolysis.. Nature.

[4] Ohh M, Park CW, Ivan M, Hoffman MA, Kim TY (2000). Ubiquitination of hypoxia-inducible factor requires direct binding to the beta-domain of the von Hippel-Lindau protein.. Nat Cell Biol.

[5] Pouyssegur J, Dayan F, Mazure NM (2006). Hypoxia signalling in cancer and approaches to enforce tumour regression.. Nature.

[6] Pollard PJ, Briere JJ, Alam NA, Barwell J, Barclay E (2005). Accumulation of Krebs cycle intermediates and over-expression of HIF1alpha in tumours which result from germline FH and SDH mutations.. Hum Mol Genet.

[7] Bardos JI, Ashcroft M (2005). Negative and positive regulation of HIF-1: a complex network.. Biochim Biophys Acta.

[8] Xenaki G, Ontikatze T, Rajendran R, Stratford IJ, Dive C (2008). PCAF is an HIF-1alpha cofactor that regulates p53 transcriptional activity in hypoxia.. Oncogene.

[9] Isaacs JS, Jung YJ, Mimnaugh EG, Martinez A, Cuttitta F (2002). Hsp90 regulates a von Hippel Lindau-independent hypoxia-inducible factor-1 alpha-degradative pathway.. J Biol Chem.

[10] Katschinski DM, Le L, Schindler SG, Thomas T, Voss AK (2004). Interaction of the PAS B domain with HSP90 accelerates hypoxia-inducible factor-1alpha stabilization.. Cell Physiol Biochem.

[11] Liu YV, Baek JH, Zhang H, Diez R, Cole RN (2007). RACK1 Competes with HSP90 for Binding to HIF-1α and Is Required for O2-Independent and HSP90 Inhibitor-Induced Degradation of HIF-1α.. Molecular Cell.

[12] Shweiki D, Itin A, Soffer D, Keshet E (1992). Vascular endothelial growth factor induced by hypoxia may mediate hypoxia-initiated angiogenesis.. Nature.

[13] Plate KH, Breier G, Weich HA, Risau W (1992). Vascular endothelial growth factor is a potential tumour angiogenesis factor in human gliomas in vivo.. Nature.

[14] Carmeliet P, Dor Y, Herbert JM, Fukumura D, Brusselmans K (1998). Role of HIF-1alpha in hypoxia-mediated apoptosis, cell proliferation and tumour angiogenesis.. Nature.

[15] Wykoff CC, Beasley NJ, Watson PH, Turner KJ, Pastorek J (2000). Hypoxia-inducible expression of tumor-associated carbonic anhydrases.. Cancer Res.

[16] Covello KL, Kehler J, Yu H, Gordan JD, Arsham AM (2006). HIF-2alpha regulates Oct-4: effects of hypoxia on stem cell function, embryonic development, and tumor growth.. Genes Dev.

[17] Staab A, Loeffler J, Said HM, Diehlmann D, Katzer A (2007). Effects of HIF-1 inhibition by chetomin on hypoxia-related transcription and radiosensitivity in HT 1080 human fibrosarcoma cells.. BMC Cancer.

[18] Wang Y, Liu Y, Malek SN, Zheng P (2011). Targeting HIF1alpha Eliminates Cancer Stem Cells in Hematological Malignancies.. Cell Stem Cell.

[19] Berchner-Pfannschmidt U, Frede S, Wotzlaw C, Fandrey J (2008). Imaging of the hypoxia-inducible factor pathway: insights into oxygen sensing.. Eur Respir J.

[20] Safran M, Kim WY, O'Connell F, Flippin L, Gunzler V (2006). Mouse model for noninvasive imaging of HIF prolyl hydroxylase activity: assessment of an oral agent that stimulates erythropoietin production.. Proc Natl Acad Sci U S A.

[21] Shibata T, Akiyama N, Noda M, Sasai K, Hiraoka M (1998). Enhancement of gene expression under hypoxic conditions using fragments of the human vascular endothelial growth factor and the erythropoietin genes.. Int J Radiat Oncol Biol Phys.

[22] Post DE, Van Meir EG (2001). Generation of bidirectional hypoxia/HIF-responsive expression vectors to target gene expression to hypoxic cells.. Gene Ther.

[23] Biechele TL, Moon RT (2008). Assaying beta-catenin/TCF transcription with beta-catenin/TCF transcription-based reporter constructs.. Methods Mol Biol.

[24] Biechele TL, Adams AM, Moon RT (2009). Transcription-based reporters of Wnt/beta-catenin signaling.. Cold Spring Harb Protoc.

[25] Shibata T, Giaccia AJ, Brown JM (2000). Development of a hypoxia-responsive vector for tumor-specific gene therapy.. Gene Ther.

[26] Ghoda L, van Daalen Wetters T, Macrae M, Ascherman D, Coffino P (1989). Prevention of rapid intracellular degradation of ODC by a carboxyl-terminal truncation.. Science.

[27] Lee YM, Jeong CH, Koo SY, Son MJ, Song HS (2001). Determination of hypoxic region by hypoxia marker in developing mouse embryos in vivo: a possible signal for vessel development.. Dev Dyn.

[28] Krishnan J, Ahuja P, Bodenmann S, Knapik D, Perriard E (2008). Essential role of developmentally activated hypoxia-inducible factor 1alpha for cardiac morphogenesis and function.. Circ Res.

[29] Harvey RP (2002). Patterning the vertebrate heart.. Nat Rev Genet.

[30] Amarilio R, Viukov SV, Sharir A, Eshkar-Oren I, Johnson RS (2007). HIF1alpha regulation of Sox9 is necessary to maintain differentiation of hypoxic prechondrogenic cells during early skeletogenesis.. Development.

[31] Provot S, Zinyk D, Gunes Y, Kathri R, Le Q (2007). Hif-1alpha regulates differentiation of limb bud mesenchyme and joint development.. J Cell Biol.

[32] Schipani E, Ryan HE, Didrickson S, Kobayashi T, Knight M (2001). Hypoxia in cartilage: HIF-1alpha is essential for chondrocyte growth arrest and survival.. Genes Dev.

[33] Schipani E (2006). Hypoxia and HIF-1alpha in chondrogenesis.. Ann N Y Acad Sci.

[34] Macfarlane CM (1997). In vitro influence of sublethal hypoxia on differentiation of the 3T3-L1 preadipose cell line and its physiological implications.. Life Sci.

[35] Sahai A, Patel MS, Zavosh AS, Tannen RL (1994). Chronic hypoxia impairs the differentiation of 3T3-L1 fibroblast in culture: role of sustained protein kinase C activation.. J Cell Physiol.

[36] Modarressi A, Pietramaggiori G, Godbout C, Vigato E, Pittet B (2010). Hypoxia impairs skin myofibroblast differentiation and function.. J Invest Dermatol.

[37] Kong X, Alvarez-Castelao B, Lin Z, Castano JG, Caro J (2007). Constitutive/hypoxic degradation of HIF-alpha proteins by the proteasome is independent of von Hippel Lindau protein ubiquitylation and the transactivation activity of the protein.. J Biol Chem.

[38] Mathieu J, Zhou W, Zhang Z, Wang AJ, Heddleston JM (2011). HIF induces human embryonic stem cell markers in cancer cells.. Cancer Research.

[39] Schulte TW, Neckers LM (1998). The benzoquinone ansamycin 17-allylamino-17-demethoxygeldanamycin binds to HSP90 and shares important biologic activities with geldanamycin.. Cancer Chemother Pharmacol.

[40] Chiarotto JA, Hill RP (1999). A quantitative analysis of the reduction in oxygen levels required to induce up-regulation of vascular endothelial growth factor (VEGF) mRNA in cervical cancer cell lines.. Br J Cancer.

[41] Pilch H, Schlenger K, Steiner E, Brockerhoff P, Knapstein P (2001). Hypoxia-stimulated expression of angiogenic growth factors in cervical cancer cells and cervical cancer-derived fibroblasts.. Int J Gynecol Cancer.

[42] Lin Z, Bazzaro M, Wang MC, Chan KC, Peng S (2009). Combination of proteasome and HDAC inhibitors for uterine cervical cancer treatment.. Clin Cancer Res.

[43] Schwock J, Pham NA, Cao MP, Hedley DW (2008). Efficacy of Hsp90 inhibition for induction of apoptosis and inhibition of growth in cervical carcinoma cells in vitro and in vivo.. Cancer Chemother Pharmacol.

[44] Barbosa MS, Vass WC, Lowy DR, Schiller JT (1991). In vitro biological activities of the E6 and E7 genes vary among human papillomaviruses of different oncogenic potential.. J Virol.

[45] Nakamura M, Bodily JM, Beglin M, Kyo S, Inoue M (2009). Hypoxia-specific stabilization of HIF-1alpha by human papillomaviruses.. Virology.

[46] Huh K, Zhou X, Hayakawa H, Cho JY, Libermann TA (2007). Human papillomavirus type 16 E7 oncoprotein associates with the cullin 2 ubiquitin ligase complex, which contributes to degradation of the retinoblastoma tumor suppressor.. J Virol.

[47] Barry SC, Harder B, Brzezinski M, Flint LY, Seppen J (2001). Lentivirus vectors encoding both central polypurine tract and posttranscriptional regulatory element provide enhanced transduction and transgene expression.. Hum Gene Ther.

[48] Yan Q, Bartz S, Mao M, Li L, Kaelin WG (2007). The hypoxia-inducible factor 2alpha N-terminal and C-terminal transactivation domains cooperate to promote renal tumorigenesis in vivo.. Mol Cell Biol.

[49] Vandesompele J, De Preter K, Pattyn F, Poppe B, Van Roy N (2002). Accurate normalization of real-time quantitative RT-PCR data by geometric averaging of multiple internal control genes.. Genome Biol.

