# Supplementary figures and images for: Assessment of Hypoxia Inducible Factor Levels in Cancer Cell Lines upon Hypoxic Induction Using a Novel Reporter Construct
Source: PLoS One. 2011 Nov 23;6(11):e27460. doi: 10.1371/journal.pone.0027460 (PMC3223176; doi:10.1371/journal.pone.0027460)

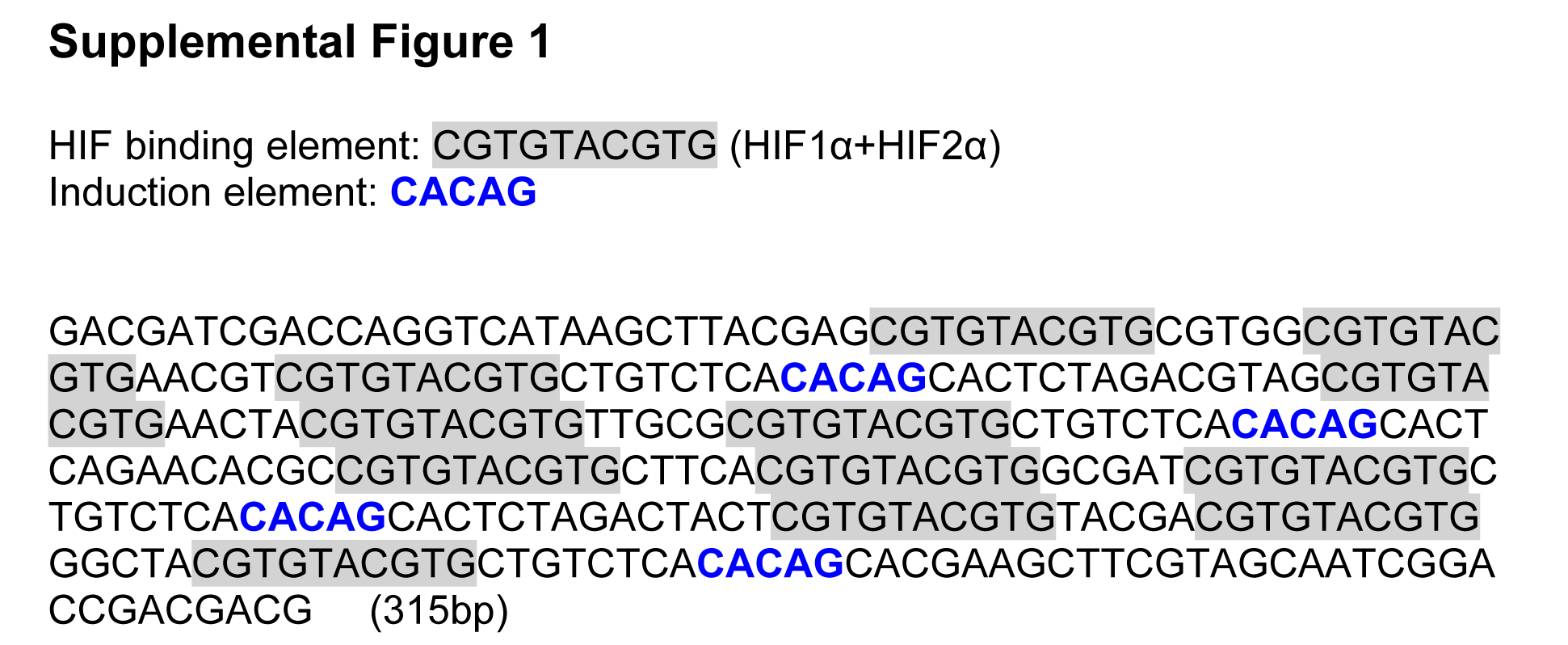

Supplement: Figure S1 — Promoter sequence of 12U-HBR construct. The minimal TK promoter is not shown. (TIF) [file pone.0027460.s001.tif]

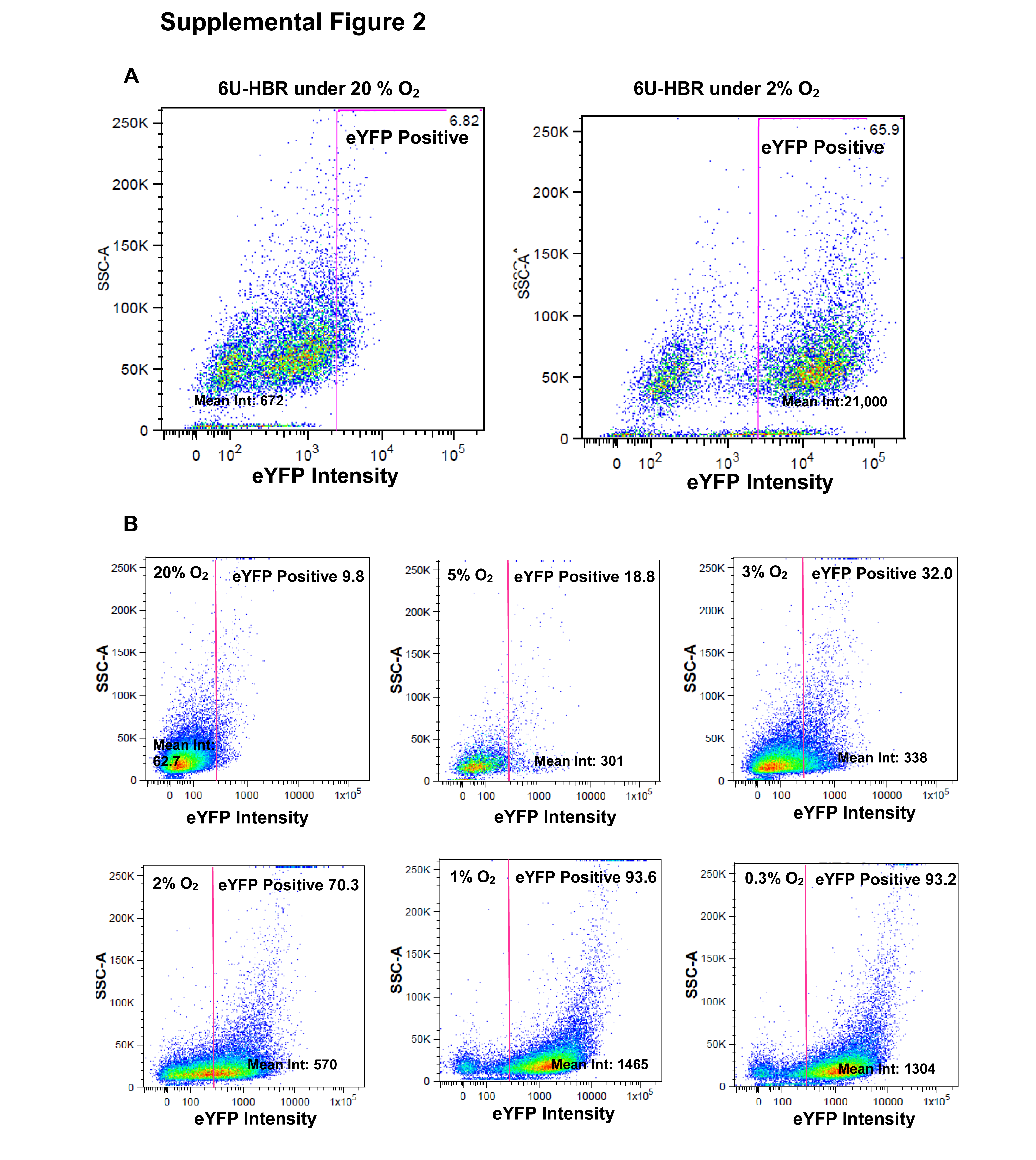

Supplement: Figure S2 — 6U-HBR HeLa cells response to hypoxia as reflected by the reporter construct. a) 6U-HBR HeLa cells turn eYFP on in 2% but not in 20% oxygen. Equal amount of cells (5×10^4^) were plated in both culture dishes and FACS analysis were performed after 2 days. The percentages of eYFP positive cells under these conditions are shown. b) The patterns of 6U-HBR HeLa cells in response to different levels of hypoxia. The percentages of eYFP positive cells and the mean intensity of each sample were shown. (TIF) [file pone.0027460.s002.tif]

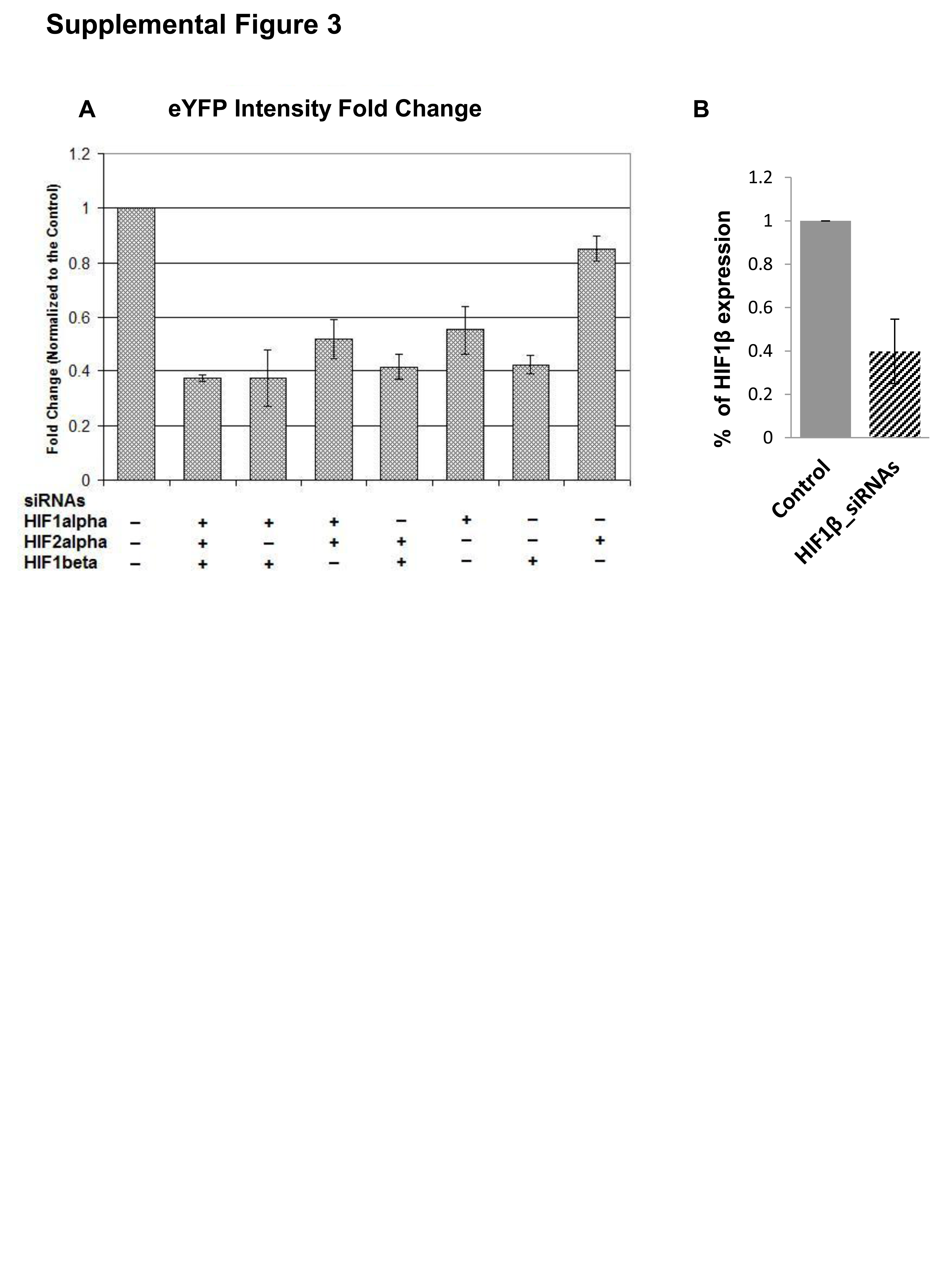

Supplement: Figure S3 — 6U-HBR construct is HIF-dependent. a) siRNAs assays against various combinations of HIFs demonstrate that the construct is HIF-dependent. HeLa cells were incubated in 2% O2 for one day after siRNA transfection; b) Validation is shown for knockdown experiments of HIF1β expression by HIF1β siRNAs. Mean and error bars are from three biological repeats. (TIF) [file pone.0027460.s003.tif]

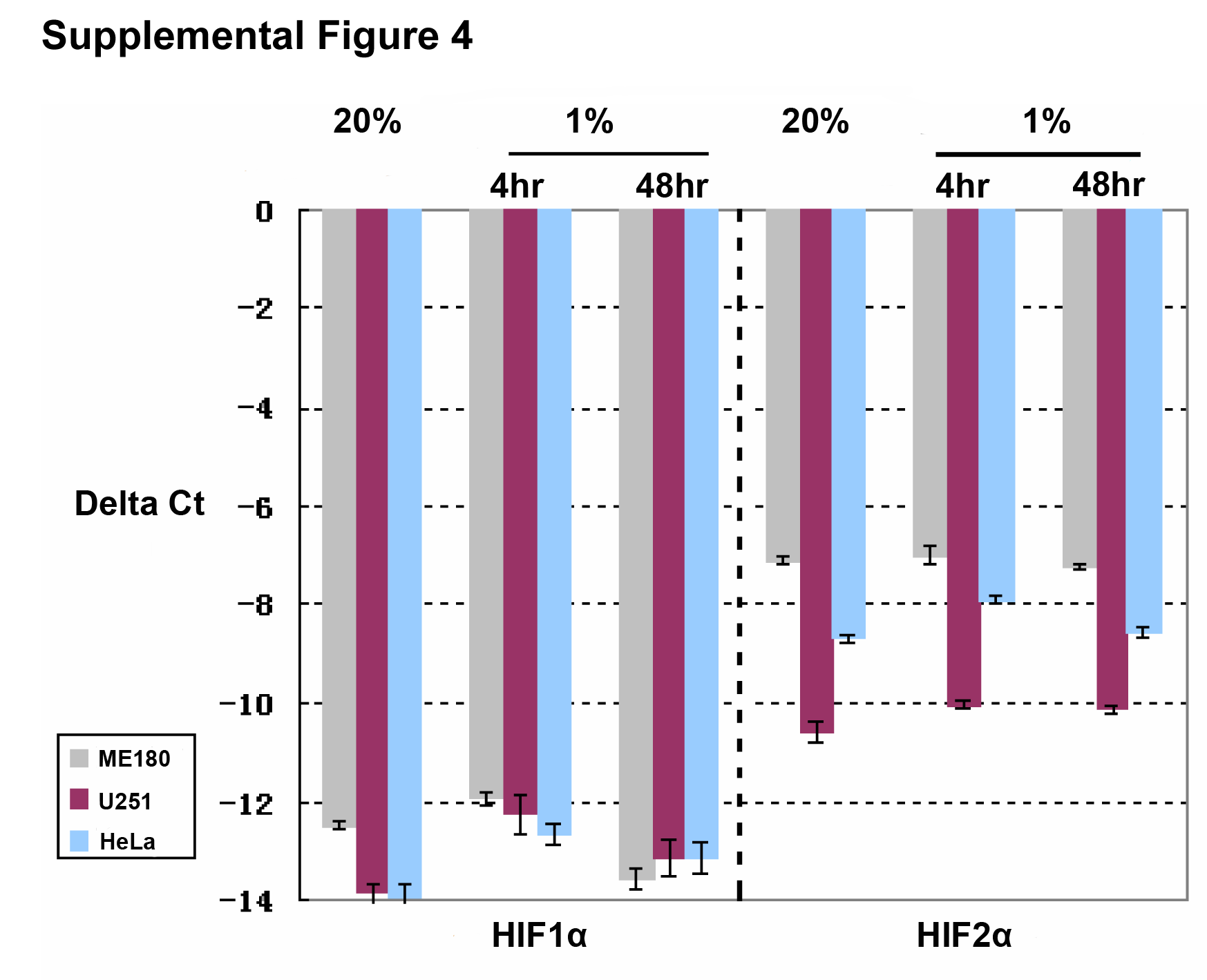

Supplement: Figure S4 — HIFα mRNA levels in ME180, U251 and HeLa cancer cell lines. Delta Ct is calculated directly from Ct values of endogenous genes (HPRT1 and YWHAZ) minus that of HIF1α or HIF2α. (TIF) [file pone.0027460.s004.tif]

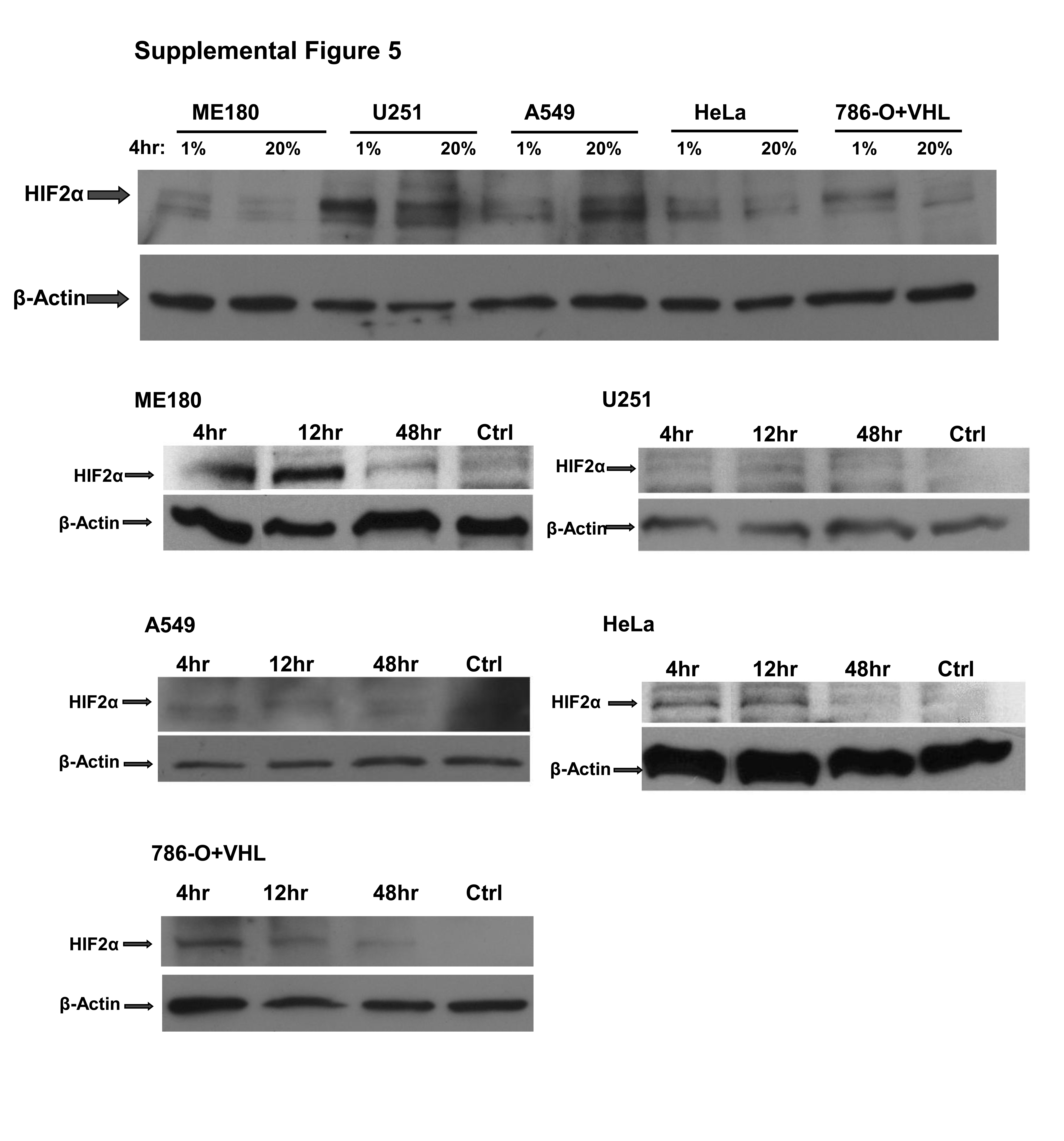

Supplement: Figure S5 — HIF2α protein levels under 1% O2 in five cell lines. a) HIF2α in ME180, U251, A549, HeLa and 786-O+VHL cells under 1% O2 for 4 hours. b) HIF2α levels in ME180, U251, A549, HeLa and 786-O+VHL cells under 1% O2 at three time points: 4 hours, 12 hours and 48 hours, compared to the levels under 20% O2 as controls. HIF2α antibody (Novus Biologicals,CO, NB100-122) was used. (TIF) [file pone.0027460.s005.tif]

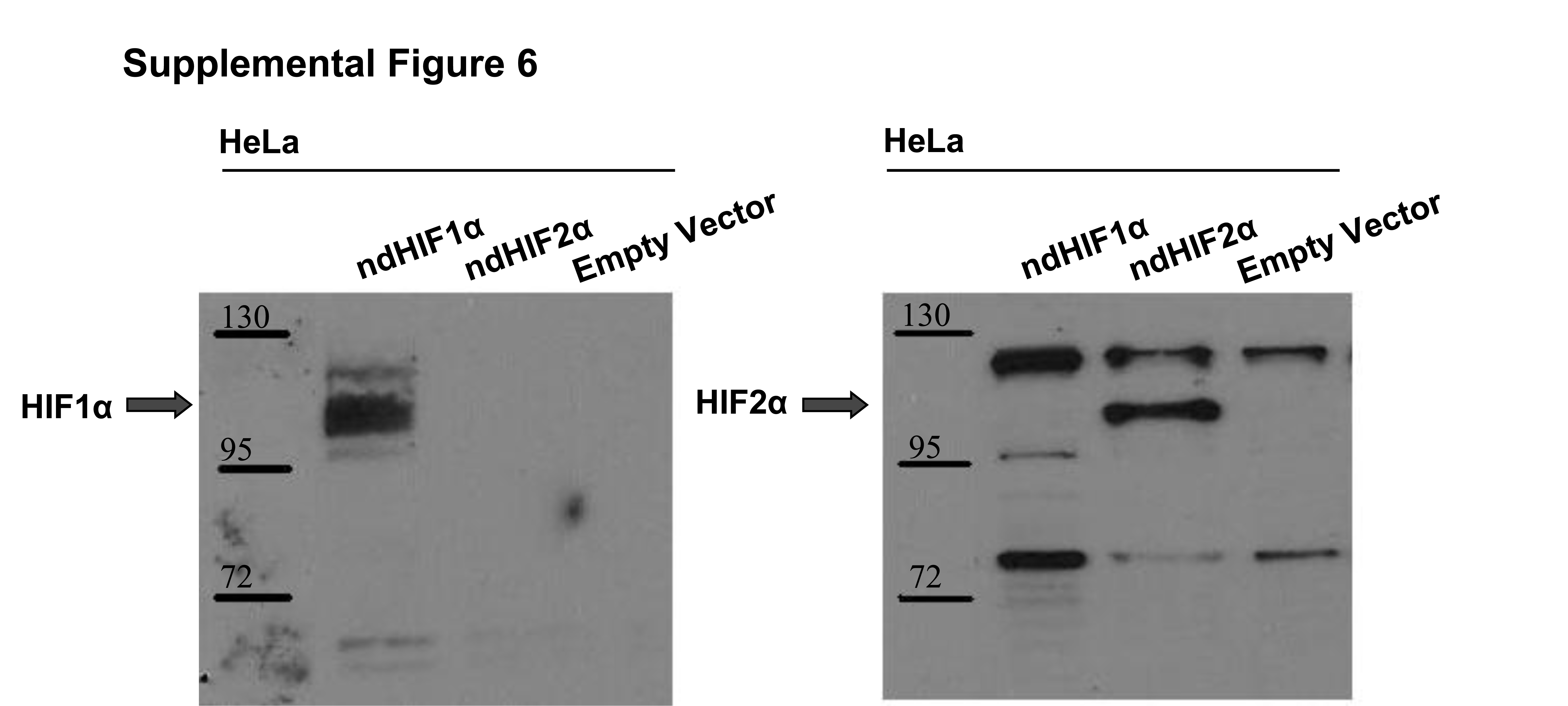

Supplement: Figure S6 — Over-expression of HIF1α and HIF2α by retroviral infection was confirmed in western blots. HIF2α antibody (Abcam, Cambridge, MA, ab20654) was used. (TIF) [file pone.0027460.s006.tif]
